# The hazards of hazard identification in environmental epidemiology

**DOI:** 10.1186/s12940-017-0296-3

**Published:** 2017-08-09

**Authors:** Rodolfo Saracci

**Affiliations:** International Agency for Research on Cancer, Lyon, France

**Keywords:** Bayes’ theorem, Conflict of interest, Hazard identification, IARC Monographs, Linearity, Model assumptions, Risk assessment, Threshold

## Abstract

Hazard identification is a major scientific challenge, notably for environmental epidemiology, and is often surrounded, as the recent case of glyphosate shows, by debate arising in the first place by the inherently problematic nature of many components of the identification process. Particularly relevant in this respect are components less amenable to logical or mathematical formalization and essentially dependent on scientists’ judgment. Four such potentially hazardous components that are capable of distorting the correct process of hazard identification are reviewed and discussed from an epidemiologist perspective: (1) lexical mix-up of hazard and risk (2) scientific questions as distinct from testable hypotheses, and implications for the hierarchy of strength of evidence obtainable from different types of study designs (3) assumptions in prior beliefs and model choices and (4) conflicts of interest. Four suggestions are put forward to strengthen a process that remains in several aspects judgmental, but not arbitrary, in nature.

## Background

Hazard identification is a major theme and challenge for environmental epidemiology, often fueling heated debate, as the recent [1] and ongoing [2] case of glyphosate carcinogenicity shows. Debate arises in the first place because the hazard identification process is inherently complex in many of its components, particularly those that depend much more on scientists’ judgment than on procedures amenable to logical or mathematical formalization. In this paper I dwell upon such components, i.e. (1) hazard and risk terminology (2) scientific questions versus testable hypotheses (3) assumptions and (4) conflicts of interest. All four are of a general nature and built-in to any system of evidence evaluation for hazard identification. For this reason they may be overlooked or underrated, remain at least in part implicit and become ‘hazardous’, namely capable of insidiously torpedoing the evidence evaluation process with the induction of false negative or false positive results or of misunderstandings on the very meaning of words used to classify an exposure as hazard. This is relevant to all evidence evaluation systems of hazards (environmental and others) such as the International Agency for Research on Cancer (IARC) procedures for identifying exposures carcinogenic for humans [3], the US Environmental Protection Agency reviews of pesticides for carcinogenic potential [4] or the International Panel on Climatic Change methodology and treatment of uncertainty [5]. It is also pertinent to possible new developments, as for example in the adaptation for environmental hazard identification of the GRADE system [6], well established in the framework of the international Cochrane collaboration for systematic reviews of interventions in clinical medicine and, more recently, public health [7]. With this in mind this paper, while reflecting an epidemiologist’s viewpoint, is written in an expository mode for a potentially wider readership.

## The four issues

### Hazard and risk

First a lexical clarification is in order, seemingly trivial but in my experience not at all obvious when communicating about hazards not only with lay people but also among scientists of different disciplines. *Hazard* is currently employed in one clearly definite meaning: an exposure, simple (a specific physical, chemical or biological agent) or variably complex (mixtures or behaviors such as tobacco smoke or night shift work) capable of causing an adverse health effect. *Risk* apart from being occasionally and improperly used as synonym of hazard (“fast driving is a risk”) has two overlapping meanings: (1) *probability* that an* event of any kind* (e.g. an adverse event such as death or favorable event such as recovery) occurs within a given time interval, say a year. This is the formal, general definition [8] (2) *probability* that a well defined *adverse event*, say cancer in a particular organ, occurs within a given time interval. This is the most common meaning. If an exposure entails in all circumstances zero risk of a specified adverse effect it is not an hazard, if it entails a non-zero risk it is an hazard: distinguishing between these two fundamental alternatives is the task of *hazard identification* (as done for example in the IARC “Monographs on the evaluation of carcinogenic risks to humans”) while estimating the size of non-zero risks is the task of *quantitative risk assessment*, for which hazard identification is the pre-requirement. The additional input of an often very imperfect knowledge of exposure distribution leads to *risk characterization* in a given population. The subsequent steps on the way to final decisions and implementation of health protecting measures applicable to the specific population are comprehensively labelled *risk management* and calls for different types of inputs, including technical, economic, ethical and political.

### Scientific questions versus testable hypotheses

Scientific questions and testable hypotheses* are very distinct concepts: questions arise all the time that can be perfectly formulated in scientific terms but at the present state of the art cannot be translated into answerable questions, namely testable hypotheses. Consider the example of mobile phones: when the first large international case-control study, coordinated by the IARC (the “Interphone” study [9]), was conducted the scientific question was, and in fact it still remains: “Is radiofrequency energy as emitted by mobile phones carcinogenic?” However at the time of the study –the early years of this century - the testable hypothesis was “Is radiofrequency energy emitted by first and second generation mobile phones producing cancer in the first ten to twelve years since onset of exposure?” To appreciate the gulf separating the scientific question from the testable hypothesis it can be recalled that in the first ten to twelve years since onset of exposure it would have been hard to confidently detect even the effect of tobacco smoking on lung cancer occurrence. The “Interphone” hypothesis was actually further restricted to the brain –the most likely organ to be affected – and the results showed a 40% elevation in the risk of glioma only in the highest category of duration of mobile phone use, with no exposure-response gradient [9].

* testability is here understood in the general sense of a tool for hypothesis verification (or, in popperian terms, “falsification”) and not in the technical sense of statistical hypothesis testing

The general distinction between scientific questions and testable hypotheses is relevant in two respects.

1. *What is actually tested and how adequately*? Any evidence evaluation only applies to the actual hypotheses tested. It may or may not be applicable, in a mediated way, to the scientific question. For instance, how far would a clear increase only in meningiomas, biologically self-limiting tumours, reply to the scientific question of carcinogenicity of radiofrequency energy? And on the exposure rather than on the endpoint side how does a commercial formulation of a pesticide reflect the effect of the key molecule under investigation? Bridging testable or tested hypotheses to scientific questions with their public health implications is an inherently “hazardous” exercise of an entirely judgmental nature, and no amount of pedantic instructions can do the job in the absence of that judgment.

Within any system in current use the weight of evidence from a single study in favor or against an exposure to be an hazard depends on the study quality. This involves two adequacy issues: methodological adequacy to test the hypothesis and adequate, namely clear and complete specification of the hypothesis as testable and distinct –as is often the case- from the scientific question. A key issue of the next stage in the process, namely, aggregation of studies considers the design typology of studies in relation to a “hierarchy of evidence” of different degrees of certainty they may produce. Randomized controlled trials are usually placed – for example in the Cochrane collaboration [10] and related approaches - at the top of the hierarchy scale and anecdotal case series and reports, if considered at all, at the bottom, with observational studies, themselves graded by type, in between. A typology of studies permits to develop tools, such as exhaustive check-lists, to assess potential biases affecting each study type, randomized trials included. These tools are indispensable at all stages of an investigation - planning, conduct, analysis and interpretation - as well as for critical “a posteriori” reviews. However to transpose typology and tools into a generally applicable hierarchy, i.e. a ranking order of studies by quality of the evidence they are assumed to provide is a jump justifiable in the realm of ideas, where the perfect study of one type is compared to the perfect studies of other types but not in the real world of empirical data available for hazard identification. Here the quality of evidence depends on how a study -whatever the type- has been implemented, a feature that can be scrutinized through a detailed quantitative analysis of biases, performed using pertinent tools such as the Cochrane Risk of Bias Tools [10]. Even a randomized trial, carefully planned like the well-known MRFIT trial on the prevention of ischemic heart disease [11] provided less clear-cut and informative results than observational studies. *One ranking order cannot fit all real life studies* and at the extreme even the evidence from a single observation may argue strongly for some peculiar cause being at work. As Henry David Thoreau said “Some circumstantial evidence is very strong, as when you find a trout in the milk” [12]. While historical experience cannot be taken as a unerring guide to the future it cannot be dismissed either: the examples of Table 1 (that could be easily extended to other diseases than cancers) show that convincing evidence of carcinogenicity in humans was reached for a number of exposures by a variety of study types.

**Table 1:** Types of studies based on which the level of convincing evidence of carcinogenicity was reached for a number of exposures^a^

Exposure	Type of study
Tobacco smoke	Cohort studies
Diethylstilbestrol	Case-control study (small)
Bis(chloromethyl)ether	Case series
Benzene	Case reports
Arsenic	Ecological study
Several chemotherapeutic drugs	Controlled randomized trials

^a^Source: [16]. Evidence rated as convincing when corresponding exactly or closely to category 1 or 2A in the current definitions of IARC [3]

To take an earlier instance, already in 1967 Richard Doll in his seminal review of human carcinogens noted that in the reported leukemia cases with occupational exposure to benzene a high proportion (some 20%) was represented by erythro-leukemia, a very rare subtype (1–2% among the cases from the general population), and concluded that this finding “strongly suggests the existence of some special etiological relationship” to benzene [13].

**Fig. 1:**
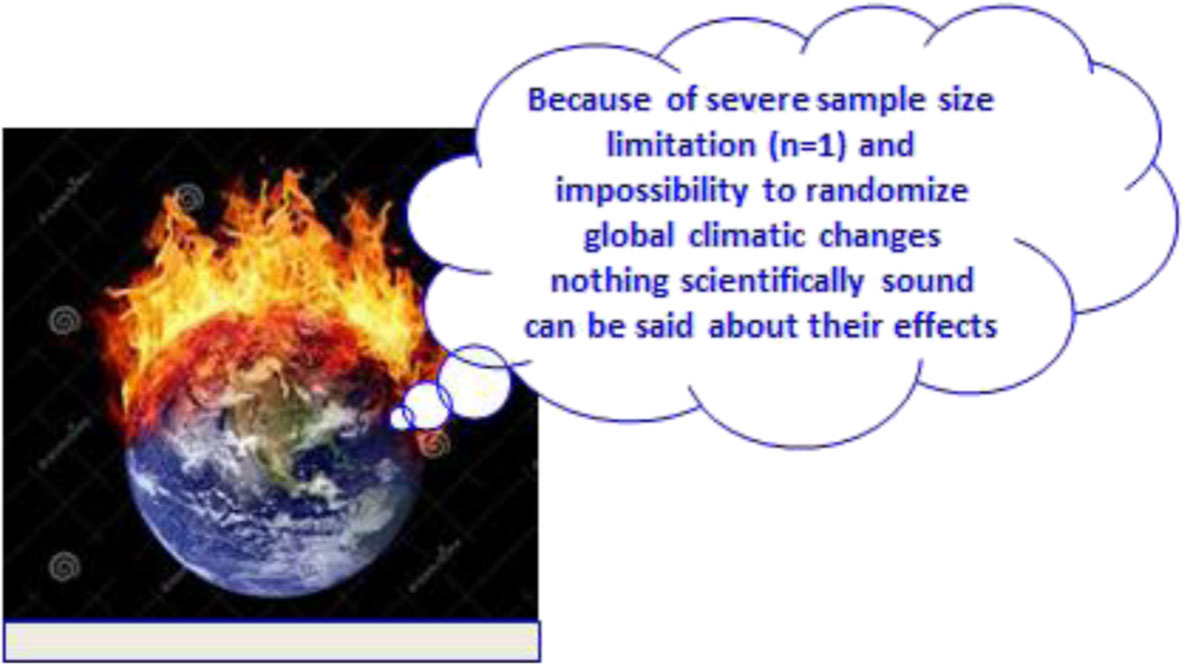
Where the boundaries lie between science and non-science?

(2) *What constitutes complete information on a hazard?.* Distinguishing the scientific question from the testable hypothesis has an important relevance for what constitutes complete information on hazard identification. This includes not only the degree of confidence (certainty) in the evidence for or against an exposure to be hazardous,as reported by any of the classification systems in use, but should also include – and this instead is almost never reported – a second information item: how the hazard hypothesis can be tested. Is a randomized trial in principle feasible or it would be unethical, illegal, unacceptable by anyone, hence unfeasible because of the toxic nature of the exposure? Are single or repeated observational studies possible, of which type and where are the potentially investigable populations? Are we dealing with a “one-off” complex and evolving exposure for which no comparative study is possible? (Fig. 1).
  One key point needs emphasizing: knowledge of what studies would be feasible cannot and must not modify the judgement of the actual evidence already available, but at the same time knowledge of what is feasible is a factual (not judgmental) element essential as an input to the subsequent decision stages of the hazard control process. It is, time scale differences apart, exactly the same situation as when a clinician has to decide what to do: the decision may be vastly different if he/she knows that it is possible to acquire further needed diagnostic information or if there is no such possibility, as is almost the rule in places with poor facilities, and he/she has to act on available evidence, good or bad as it may be. And in public health as in clinical medicine no action, leaving things to their course, is itself an action. More than fifty years ago Bradford Hill had already made these points crystal clear (my italics) [14]: “The evidence is there to be judged *on its own merit* and the judgment (in that sense) should be utterly independent of what hangs upon it- or who hangs because of it ….All scientific work is incomplete – *whether it be observational or experimental*. All scientific work is liable to be upset or modified by advancing knowledge. That does not confer us a freedom to ignore the knowledge we already have, or to postpone the action that appears to demand *at a given time*”.

### Assumptions

Simple and less simple assumptions are an integral part of any scientific analysis the human mind can perform, hazard identification being no exception. One trait of assumptions is to be often completely or partially not explicit. Two assumptions are of particular relevance in hazard identification.

1. *Prior beliefs.* Any hypothesis-testing analysis of empirical data is inevitably approached by every researcher with some “a priori’ belief”, however weak. The direction and strength of this belief may drastically change the interpretation of empirical data analysis from epidemiological studies, as highlighted by a simple scenario.

Imagine that validated past experience tells that when a set of, say, three high quality cohort studies with no detected biases turn concordantly “positive” for a carcinogenic effect of the exposure under study a true carcinogen is correctly recognized in 80% of instances (again for simplicity assume “positive” meaning a 50% increase in confounders-adjusted risk at one target organ, say lung, statistically significant at 5% level). However nothing being perfect in this world, the very same concordant result may turn out falsely positive for a minority of non- carcinogenic agents, say 10%. Two researchers, John and Jane, well aware of this past experience, are now asked to assess the evidence of possible carcinogenicity of a new molecule, for which (a) there is a generic suspicion of carcinogenicity based on structural chemical analogy but the scarce, poor quality experimental data are essentially uninformative, and (b) three good quality cohort studies have become available showing positive concordant results for the same target organ. John is a believer that an agent should “a priori” be regarded as non-carcinogenic until the contrary is proven in humans and, prior to seeing any results, he assigns a 95% probability, namely 19 to 1 chances (odds), in favor of the molecule being non-carcinogenic. This may seem a legitimate assumption, if anything too pessimistic, not only to the originator or producer of the new molecule but to anyone contemplating the universe of all molecules from which one would be picked-up at random and examined for carcinogenicity in humans. After seeing the positive results of the epidemiological studies John concludes that, notwithstanding their positivity, the chances are slightly more than 2 to 1 that the new molecule *is not carcinogenic*. To reach this conclusion he has simply applied the Bayes theorem [15] to combine his prior belief with the empirical results. Jane instead considers, again before seeing any results, that for the new molecule there was a generic suspicion of carcinogenicity and turns her attention to the universe of agents included in the IARC Monographs programme [16], in which agents are examined only when there is evidence of human exposure and some scientific evidence or suspicion of carcinogenicity. In the programme 119 agents were classified as carcinogenic for humans (category 1) and 81 as probably carcinogenic (category 2A), altogether 200 out of a total of 998 examined (about 20%). As a consequence Jane assigns to the new molecule a 20% probability of being carcinogenic (or, conversely, an 80% chance of being non-carcinogenic, in contrast to the 95% by John). After seeing the positive results of the epidemiological studies and applying Bayes’ theorem she concludes that the chances in favor of the chemical *being carcinogenic* are 2 to 1, a diametrically opposite conclusion to John’s. The conclusions would be even more problematic had the results available to Jane and John been less clear-cut. Imagine only two out of three cohort studies positive, a finding shown by previous validated experience to correctly identify a carcinogen in only 60% of the instances (and not 80% as in the in the initial scenario) and yielding a 15% of falsely positive results. John would conclude that the chances for the molecule *not to be carcinogenic* are 3.8 to 1, almost settling the issue and discouraging further investigations whereas Jane would remain totally in doubt and urge further studies because for her the chances are 1 to 1 that the molecule *is or is not carcinogenic*. Two implications follow:

- Prior beliefs matter all the time but carry more weight when the empirical evidence from the available studies is unclear or weak;
- Like John and Jane we combine prior beliefs with fresh empirical results but unlike them we generally do it not through formal procedures like the Bayes theorem but informally and judgmentally, and that may involve very different logical paths (Bayes-like or not) of different rigor. Crucially our assumptions do not usually have numerical expression and would be even hard to make explicit, variedly based as they are not only on previous knowledge, specific to the issue at hand and general, but also on personal conscious and unconscious inclinations and idiosyncrasies. Yet, as the scenario shows in an elementary form, prior beliefs may have major influence on result interpretation.

(2) *Thresholds.* A second frequent and important set of assumptions concerns the stage following hazard identification, namely quantitative risk assessment. Over the last half a century a large number of papers has been written exploring the forms and the regulatory implications of exposure-response (dose-response) curves, a key and permanently debated issue being the presence or absence of a threshold of exposure below which no response occurs [17, 18]. This “fires back” into hazard identification, because if a threshold exists an agent would qualify as “hazard” only above the threshold level of exposure, a fact of great public health importance.. The relevant question is not whether thresholds for adverse effects, acute or chronic, exist for an individual: their existence makes sense biologically and for specific effects they may be demonstrated experimentally. However what matters in hazard identification as an information input to hazard control is not the threshold for an individual but the human population threshold, below which no individual will experience the adverse effect. Whether a population threshold exists depends on the thresholds distribution in the free living human population, the cumulative distribution being nothing else than the dose-response function. Defining this curve requires assumptions involving:

- Sampling variability. The shape of the curve fitted to the observed points may show no indication of a threshold, yet a threshold value may fall within the confidence interval around the curve, leaving open the question of the population threshold; moreover the values of the interval depend on the assumed level of confidence (e.g. 95% or 80% or 50% confidence interval). Similarly testing the fit of a specified form of the curve (e.g. linear, quadratic, probit, logit) requires choosing by assumption the value of the alfa error (5% or 10% or 20%) for the test. These assumptions have the advantage of being explicit - as they are in any other statistical analysis - but they remain assumptions.
- Model specification. The no threshold, linear assumption has been most often employed with human data, subject to recurrent arguments of support [19] or critique [20]. It may be derived from more complex, biology inspired, models like the multistage carcinogenesis model or be directly adopted on three grounds:

- Parsimony*.* Any dose-response relationship for environmental toxic agents must have as a minimum a linear component, which therefore represents the most parsimonious default assumption;
- Falsification failure. Falsifying the assumption is hampered by sampling variability preventing the detection with any confidence of curvilinear departures from the model;
- Addition-to-background. The argument, originally introduced for cancer [21, 22], rests on the fact that in human populations almost all diseases exhibit a baseline (background) incidence even in the absence of specific agents, for example asbestos fibers investigated in connection to lung cancer. Not only does lung cancer occur in people with no documentable exposure to asbestos but so does, albeit rarely, even an uncommon disease as mesothelioma. Similarly liver angio-sarcoma can rarely occur in people with no exposure to vinyl-chloride. A background incidence implies that the population threshold – if one exists – has already been exceeded and a positive dose-response gradient applies. Adding a small dose of the agent under study, asbestos fibers, which shares components of mechanism of action (e.g. oxygen radicals production) with known and unknown agents causing the background incidence, will simply increase the lung cancer incidence proportionately to the added dose. Formally the dose-response curve treated as a mathematically “smooth function” is linearized at low added dose with the slope of the line estimated by the first derivative. Two assumptions are then possible: either it can be excluded that the agent under study and the background agents share some mechanistic components, and linearity is not assured, or it cannot be excluded, and linearity follows. The latter assumption is much less demanding and more plausible than the former given “the multifactorial nature of disease, that is…a single adverse effect might result from multiple mechanisms that can have multiple components” [23].

Beyond the issue of linearity the main point of this whole discussion is to emphasize the ubiquity, complexity and potential impact of assumptions.

### Conflicts of interest

In the 2009 definition by the USA Institute of Medicine **“**Conflicts of interests are defined as circumstances that create a risk that professional judgements or actions regarding a primary interest will be unduly influenced by a secondary interest” [24]. “Circumstances that create a risk” is exactly what an hazard is. As a chemical or biological hazard endangers health so conflict of interest endangers a healthy, namely correct, scientific judgment: it cannot be ignored [25] and it has to be dealt with within the process of hazard identification. Several approaches can be used to (in current terminology) “manage” conflicts of interest: some, like David Michaels [26], believe that it cannot be managed at all and that the only effective measure is to tackle it at the root and eliminate it. In practice this however clashes with the widening extent of conflicts of interest within contemporary societies that appear in this respect schizophrenic: on one side prevalent societal pressures promote the mixing of conflicting interests, for example by developing loosely supervised public-private partnership, allowing academics to take up consultant roles with private corporations, favoring “revolving door” practices, while on the other side societies at large vocally pretend to have “independent” experts readily available when it comes to health or environmental problems. Management of conflicts of interest can take place at different levels:

1. *Prevention of conflicts* is the best option as it removes the conflict, either because, for example, a professional with conflicts is not accepted as a member of an expert committee or (s)he is accepted on the condition of giving-up (or having given-up for a suitable interval of time) her/his secondary interests.
2. *Prevention of effects*. It aims at preventing the conflict effects, namely a distortion of the scientific judgment on a health hazard, by –for example- limiting the function of the committee member to providing information on specific technical points with no other possibility of intervention. This is the approach that IARC has adopted. Participants in the Working Group meetings of the Monographs act in different roles [3], full members, representatives of national or international health agencies or simply observers, admitted on the condition that they abstain from influencing the proceedings: only full members, with no declared or otherwise known conflict of interest, are entitled to vote.
3. *Control of effects*. If none of the measures at the first two levels has been taken two kinds of possible effects need to be taken into account. First a demonstrable distortion of the hazard evaluation may have occurred and one is confronted with a case of major professional fault or misconduct or even fraud: these cases do occur but are clear cut and, fortunately, uncommon. They are in principle liable to legal or deontological prosecution. Second, and a reason for much wider concern, less detectable distortions may have occurred arising from the influence that a conflict of interest may exercise on the many steps in the evaluation process that rely on subjective judgment of each scientist, as discussed in the previous sections of this paper. An uncontrolled conflict of interest is like a known but uncontrolled confounder, say smoking in an occupational study: we can only conjecture about its effect but we cannot as a rule accord to studies affected by uncontrolled conflicts the same quality of evidence of studies not so affected. It is disturbing but unavoidable as results from well conducted surveys of studies have repeatedly shown, in environmental [26] clinical [27] and nutritional [28] epidemiology how hazards and harms are underrated and benefits overrated when studies are, typically, commissioned by the producers of the agents under investigation.

Conflicts of interest shifts the view from the technical to the societal level and to politics. Like any other prevalent hazard they raise a key question: how can their occurrence be reduced? Unfortunately the common answer, cast essentially in terms of ethics, is seriously inadequate and leads directly into the dead-end of relying almost exclusively on researchers honesty, however important this may be. Invoking honesty is easily and unanimously agreeable but at the same time largely irrelevant as the key issue is not the personal ethical choices of researchers (on which colleagues cannot and should not have any right) but the public, societal relevance of possible erroneous scientific judgments as a result of those choices. Hence conflicts of interest should be seen for what they primarily are, dysfunctional components of contemporary research systems which hamper and slow down the attainment of scientifically valid evidence. By making research less efficient they increase its costs to society. In this perspective the treatment of conflicts of interest depends primarily on societal, namely political, choices, which should promote and guarantee objective material and institutional conditions of independence of researchers in public national and international institutes and organizations. Conversely this points the finger at politics, when it places private interests ahead of the public good, as one major hazard for hazard identification.

## Conclusions

Out of this review that focused on the problematic aspects of hazard identification I venture, on a positive key, four suggestions.

1. A specification of what further epidemiological studies may be *actually possible* (if any) ought to be part of any information package on hazard identification, as this item is critical for public health decisions. The scientists in a position and qualified to provide such information are the same involved in the hazard identification process.
2. Assumptions are inevitable and constitutive of any analysis and interpretation of factual data which, contrary to received unwisdom, never “speak by themselves”. The way of turning assumptions into factors contributing to a correct identification of hazard is to have scientists with a variety of competences and perspectives working together (jointly and in specialty subgroups of ‘optimal’ size, say 5 or 6 persons [29]) and vigorously debating the respective positions *under objectives conditions free of any interest extraneous to the pursuit of scientific truth usable for public health*. Epidemiologists should be pro-active in promoting political decisions crucial to the establishment and maintenance of such objectively and openly documentable conditions.
3. Any evidence evaluation system is necessarily evolving in time. Important methodological developments are taking place in epidemiology on causal thinking [30, 31] and on combining the evidence from different sources (including data from laboratory experiments) by such approaches as ‘triangulation’ [32] or cellular pathway perturbation [23]: they should not be ignored and their implications for evidence evaluation systems need to be critically examined.
4. It is of much interest to explore, as in current research on the GRADE system [6] for environmental epidemiology, possible ways of disciplining and translating scientists’ judgment into formalized procedures (some might in theory even be automated): yet these need to be accompanied by equally formal validation exercises, lest the known subjectivity of an individual scientist’s judgment is replaced by unknown performance characteristics of procedures intended to be more objective.

## Abbreviations

GRADE: Grading of Recommendations Assessment, Development and Evaluation
IARC: International Agency for Research on Cancer
